# Efficacy and safety of culture-expanded, mesenchymal stem/stromal cells for the treatment of knee osteoarthritis: a systematic review protocol

**DOI:** 10.1186/s13018-019-1070-8

**Published:** 2019-01-25

**Authors:** Meredith Harrison-Brown, Corey Scholes, Kholoud Hafsi, Maimuna Marenah, Jinjie Li, Fadi Hassan, Nicola Maffulli, William D. Murrell

**Affiliations:** 1 Emirates Integra Medical and Surgery Centre, Dubai, United Arab Emirates; 2 EBM Analytics, Sydney, NSW 2065 Australia; 3 Good Hope Hospital, University Hospitals Birmingham NHS Foundation Trust, Birmingham, UK; 4 Department of Musculoskeletal Disorders, University of Salerno School of Medicine and Dentistry, Salerno, Italy; 5 Queen Mary University of London, Barts and the London School of Medicine and Dentistry Centre for Sports and Exercise Medicine, Mile End Hospital, London, England; 6 Emirates Healthcare, Dubai, United Arab Emirates; 7 Department of Orthopaedic Surgery, Landstuhl Regional Medical Center, Landstuhl, Germany

## Abstract

**Background:**

Osteoarthritis is a progressive multifactorial condition of the musculoskeletal system with major symptoms including pain, loss of function, damage of articular cartilage and other tissues in the affected area. Knee osteoarthritis imposes major individual and social burden, especially with the cost and complexity of surgical interventions. Mesenchymal stem/stromal cells have been indicated as a treatment for degenerative musculoskeletal conditions given their capacity to differentiate into tissues of the musculoskeletal system.

**Methods:**

A systematic search will be conducted in Medline, Embase, Cochrane Library, Scopus and relevant trial databases of English, Japanese, Korean, German, French, Italian, Spanish and Portuguese language papers published or in press to June 2018, with no restrictions on publication year applied. References will be screened and assessed for eligibility by two independent reviewers as per PRISMA guidelines. Cohort, cross-sectional or case controlled studies will be included for the analysis. Data extraction will be conducted using a predefined template and quality of evidence assessed. Statistical summaries and meta-analyses will be performed as necessary.

**Discussion:**

Results will be published in relevant peer-reviewed scientific journals and presented at national or international conferences by the investigators.

**Trial registration:**

The protocol was registered on the PROSPERO international prospective register of systematic reviews prior to commencement, CRD42018091763.

**Electronic supplementary material:**

The online version of this article (10.1186/s13018-019-1070-8) contains supplementary material, which is available to authorized users.

## Background

Osteoarthritis (OA) is a progressive condition affecting the articular cartilage and underlying subchondral bone, leading to significant pain and limitations in movement [1]. Knee OA is the most prevalent form of arthritis worldwide and is one of the leading causes of disease and disability amongst aging populations [2]. Recommended treatments for Knee OA can improve symptoms in many patients [3] but do not modify the underlying degeneration of the articular cartilage and alterations in architecture of the surrounding tissue. Emerging treatments derived from cellular products including platelet-rich plasma, bone marrow aspirate and mesenchymal stem/stromal cells (MSCs) have been proposed as minimally invasive alternatives to conventional therapies [4]. In particular, MSCs have been indicated as a promising treatment for degenerative musculoskeletal conditions given their anti-inflammatory properties and capacity to differentiate into osteochondral tissues [4–7].

MSCs can be obtained from the stroma of various tissues, including bone marrow, umbilical cord blood, adipose tissue, peripheral blood and synovium, and expanded in culture to increase yield and enhance desired functional properties [8]. The optimal choice of tissue source is based on considerations of patient safety, ease of access, yield and indications of functional improvements in preclinical and early clinical studies [7]. Evidence obtained in vitro and in animal models indicates that MSCs from different tissue sources differ regarding their cell surface protein expression and capacity to differentiate into specific cell types [9–13]. Thus, it is not currently clear whether the source of cells has a substantial impact on functional or structural outcomes following injection into osteoarthritic knees.

There are a large number of preclinical studies reporting a beneficial effect of MSCs on cartilage degeneration and injury, ranging from mouse [14, 15], rabbit [16–18], guinea pig [19], horse [20], goat [21], to pig models of OA [22, 23]. However, the degree of methodological heterogeneity and limitations in translational relevance for particular animal models of arthritis have complicated interpretations of results [24–26]. Nonetheless, a growing number of clinical studies indicate that mesenchymal stromal cells have the potential to reduce pain; increase joint mobility, walking ability and cartilage/meniscus growth and repair tissue extension over the subchondral bone [5, 27]. In addition, a number of studies have reported no serious adverse events as a result of MSC treatment [5, 28]. However, it is not clear whether these outcomes have been examined consistently across studies.

Considering the aforementioned lack of clarity regarding cell source, methodological factors, clinical translation and outcome measurement, a systematic review is required to synthesize and evaluate the quality of the available evidence regarding the safety and efficacy of mesenchymal stem/stromal cells for knee OA. The primary objective of this review is to establish in patients or animal models of knee osteoarthritis treated with culture-expanded mesenchymal stem/stromal cells from adipose tissue, bone marrow or synovium, with or without adjunct nonoperative therapies, the clinical, structural and functional outcomes of treatment, as well as the incidence and severity of adverse events. The secondary objective of this review is to identify study, measurement and other methodological characteristics associated with treatment outcomes.

## Methods

The protocol was registered on the PROSPERO international prospective register of systematic reviews, registration number CRD42018091763. The systematic review follows the Preferred Reporting Items for Systematic Review and Meta-analysis (PRISMA) statement [29] and protocol (PRISMA-P) guidelines [30].

### Eligibility criteria

Relevant characteristics for included studies were determined using the PICOS (Population, Intervention, Comparison, Outcomes, Study Design) framework for formulating the research question and defining eligibility criteria for the literature search [31]. Characteristics for preclinical and clinical studies are presented separately as follows:

#### Population (inclusion/exclusion criteria)

All animal models of knee osteoarthritis will be considered for review, without exclusions relative to specimen sex, activity level or age. Studies will be excluded where knee (‘stifle joint’) osteoarthritis is secondary to another condition under examination (e.g. joint instability, fracture or other condition). Clinical studies involving patients diagnosed with radiographic evidenced osteoarthritis will be considered for review, without exclusions relative to sex or activity level. Articles will be excluded from analysis if they include paediatric cases (aged under 18 years at diagnosis).

#### Intervention

Studies will be included if they involve the use of culture-expanded, mesenchymal stem/stromal cells from any source delivered by intra-articular injection. Studies will be excluded if they report the delivery of cells during surgical procedures or include other cell populations in the injected concentrate.

#### Comparators

Comparators considered will include placebos, conventional non-operative therapies including steroid injections, exercise and NSAIDs and cases unaffected by knee osteoarthritis.

#### Outcomes

For preclinical research, studies including outcomes relevant to human osteoarthritis including histological appearance of cartilage and bone, results of noninvasive imaging and measurements of pain and function will be included. Biochemical analyses with unclear relevance to human OA will not be included in the review. For clinical research, studies reporting any outcomes relevant to the efficacy and safety of MSC injection will be included in the analysis. Particular attention will be paid to validated measures of patient-reported outcomes.

#### Study designs

Observational studies (cohort, cross-sectional and case-controlled prospective or retrospective studies) or randomized controlled trials (RCTs) comparing outcomes of culture-expanded MSC treatment with other modalities at any follow up period will be included. Systematic reviews will be used to source additional primary materials but will not be included in the analysis. The results of meta-analyses will be included as a study in the analysis if they meet the remaining inclusion criteria. English, Japanese, Korean, German, French, Spanish, Italian and Portuguese language papers in publication will be included, with no restrictions on publication year.

### Information sources

A systematic search will be conducted in Pubmed, Medline, Embase via Ovid SP, Cochrane Library and Scopus via EBSCO and relevant clinical trials databases of English language papers in publication as of June 2018, with no restrictions on publication year applied. (EBSCO, AMED, CINAHL, EMBASE, Cochrane, LILACS, MEDLINE, PEDro, Scielo, Scopus & Web of Knowledge.) Secondary searching of reference lists of key articles and grey literature will be undertaken in order to identify any additional studies potentially missed in electronic search. Active researchers in the field will be contacted to ensure relevant references have been captured.

### Search strategy

**Fig. 1:**
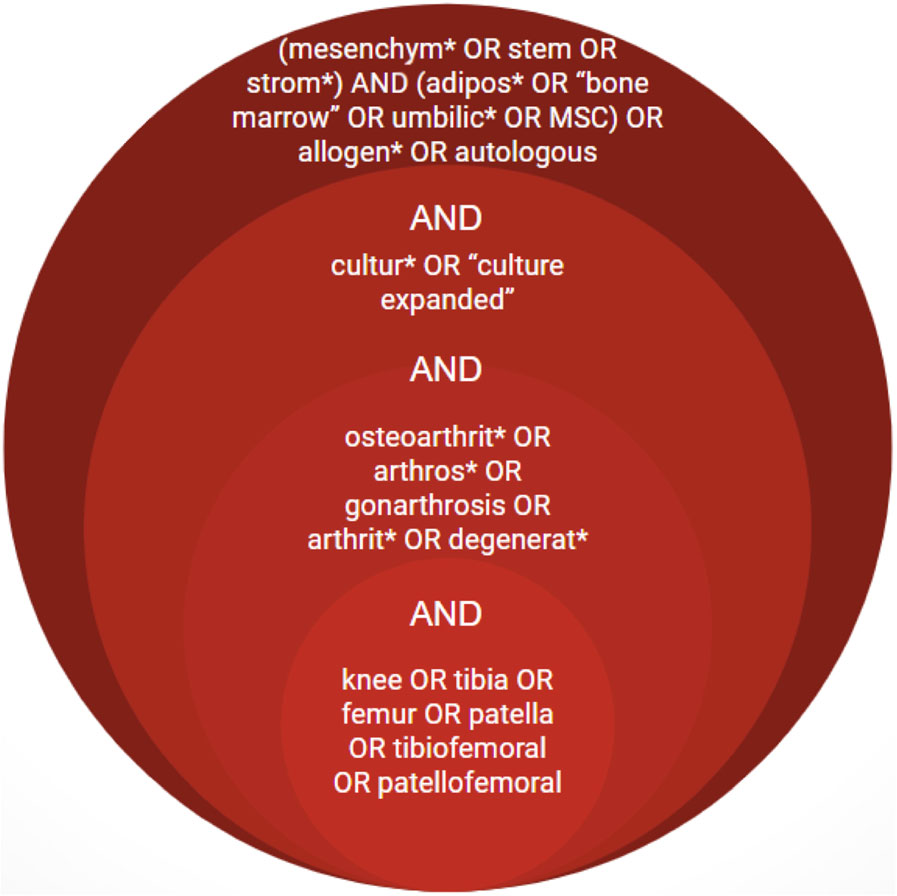
Search strategy for the systematic review

To permit the search to return other primary studies that were not included to the published reviews, medical subject headings (MeSH) terms and keywords such as systematic review, review and meta-analysis will be excluded. The following are the main key domains: (1) anatomical region, (2) pathology and (3) intervention (Fig. 1). Keywords within concept areas will be mutually inclusive (via ‘OR’ operator) and will be combined with the other key areas using an ‘AND’ operator. The search will be comprised of the following components, which will be performed individually prior to filtering for duplicate records and preliminary analysis:

1. *Anatomical region*: knee OR tibia OR femur OR patella OR tibiofemoral OR patellofemoral
2. *Pathology*: osteoarthrit* OR arthros* OR gonarthrosis OR arthrit* OR degenerat*
3. *Intervention*: (mesenchym* OR stem OR strom*) AND (adipos* OR ‘bone marrow’ OR umbilic* OR MSC) OR allogen* OR autologous AND (cultur* OR ‘culture expanded’)

The search strategy will be adjusted for application to other databases as appropriate. Search results will be supplemented by drawing relevant articles from the following:

1. Reference lists from included studies, prioritizing systematic reviews and meta-analyses
2. Clinical trial reports from Cochrane Central Register of Controlled Trials, Australia and New Zealand Clinical Trials Register, Clinicaltrials.gov, World Health Organization International Clinical Trials Registry

### Study records

The study search and selection process will be based on the four-phase PRISMA flow process [32] for identification, screening, assessment of eligibility and inclusion of studies for the systematic review. A web-based bibliographic software package (Paperpile LLC, Vienna, Austria) will be used for data management. Citations and abstracts identified during the study search will be imported to the bibliographic software and duplicates removed. The study selection process will be performed independently by two reviewers. Title and abstract screening will be performed and full text files will be retrieved and uploaded to the reference software. Eligible studies will be identified for inclusion in the review. Data extracted and synthesized by the two independent reviewers will be the following: author names, publication years, design of the included primary studies, inclusion criteria for primary studies, group intervention and comparison of the primary studies, tools used for outcomes assessment, the outcomes of interest and references of the primary studies. Customized forms will be used for assessment of eligibility during the selection process and extraction of data. Consensus for inclusion and data extraction will be established amongst co-authors prior to review commencement, with study eligibility and data extraction forms piloted by each reviewer prior to use. Where agreement for study inclusion or data extraction is unable to be reached by the two reviewers, a third reviewer from the study team will be consulted.

### Data items

Study parameters, population characteristics, treatment factors and outcomes will be extracted from included animal and human studies corresponding to the relevant items in Additional file 1.

## Outcomes

For preclinical studies, outcomes considered will include clinically relevant outcomes such as mortality, morbidity and adverse events. Structural outcomes considered include results of histological analyses, including grading of pathology according to the Osteoarthritis Research Society International (OARSI) histopathology initiative guidelines for specific animal models [33–37], and other commonly used measures such as the Grading of Recommendations Assessment, Development and Evaluation (HHGS)/Mankin score and its modifications, the O’Driscoll and Pineda scores [38]. Outcomes of noninvasive imaging including cartilage thickness [39], presence of osteosclerotic lesions or intraosseous cysts [40] visible on MRI will also be included for analysis. Functional outcomes considered for analysis will include behavioural and mechanical measures of nociception and gait analysis such as hind paw weight as appropriate to specific species [25].

For clinical studies, outcomes considered for analysis will include clinically relevant outcomes, such as mortality, morbidity and adverse events, classified as per the US Department of Health and Human Services Common Terminology Criteria for Adverse Events [41]. Structural outcomes will include results of arthroscopic evaluation, specifically ratings of severity such as the International Cartilage Repair Society (ICRS) clinical cartilage injury classification system [42, 43], and Oswestry Arthroscopic Score (OAS) [44]. Also considered will be the results of medical imaging, including ratings of x-rays such as the Kellgren-Lawrence (KL) Classification of Osteoarthritis [45] and ratings of pathology via magnetic resonance imaging such as the OMERACT Knee Inflammation MRI Scoring System (KIMRISS) [46], the Boston Leeds Osteoarthritis Knee Score (BLOKS) [47], the MRI Osteoarthritis Knee Score (MOAKS) [48] and the Whole-Organ Magnetic Resonance Imaging Score (WORMS) [49]. Results of histological analyses considered for analysis include grading systems such as the HHGS [50] and the OARSI Cartilage Histopathology Assessment System [51]. Patient-reported outcomes considered for review include validated measures of treatment response [52, 53], including measures of knee function, pain, quality of life and patient satisfaction, such as the Western Ontario and McMasters Universities Osteoarthritis Index (WOMAC) [54], the Knee injury and Osteoarthritis Outcome Score (KOOS) [55], Knee Pain Scale (KPS) [56] and visual analogue scales (VAS). Objective functional outcomes including strength, range of motion, locomotion, gait and proprioception will also be examined if reported in included studies.

### Risk of bias

The Systematic Review Centre for Laboratory Animal Experimentation (SYRCLE) risk of bias tool will be applied to pre-clinical (animal) studies [57]. This is an assessment tool adapted from the Cochrane risk of bias tool for randomized controlled trials with human participants [58] and the two tools display significant overlap. Independent scoring of risk of bias for included studies will be performed by two reviewers, with consensus reached by discussion. The ROBINS-I (‘Risk of Bias In Non-randomized Studies - of Interventions’) tool [59] will be used to assess the observational studies eligible for inclusion. Potential risks will be assessed over seven bias domains: baseline confounding, participant selection, classification of intervention, deviations from intended intervention, missing data, outcomes measurement and reporting [59, 60]. For any randomized trials, the RoB2.0 tool will be used to rate risk of bias [61]. An overall risk of bias judgement will be determined as either low, moderate, serious or critical risk of bias or no information for each specified outcome. Where more than one outcome of an included study is to be assessed, the risk of bias across the seven domains will be repeated for each key outcome, and a risk of bias judgement will be reported for all outcomes.

### Data synthesis and meta-analysis

Data synthesis and meta-analysis will be performed separately for clinical and pre-clinical studies, following the guidelines published by Shamseer et al. [30] and Hooijmans et al. [62], respectively. Where the same outcome has been reported across a sufficient number of studies, a quantitative synthesis will be conducted. Data from included studies will be loaded into Review Manager (v5.3) and heterogeneity index (I-squared) will be calculated. Given anticipated heterogeneity amongst studies, a random-effects meta-analysis followed by subgroup analyses will be performed if deemed appropriate. Subgroups chosen for analysis will include the different tissue sources of MSCs (specifically bone marrow vs adipose vs peripheral blood vs synovium) and autologous vs allogeneic cells. Results of meta-analyses will be presented graphically via forest plots, and summary effects will be presented. Publication bias will be assessed using funnel plots with standard error. Where required, mirroring of low sample studies will be used to enable visualization. Where quantitative synthesis is not appropriate, the extracted data will be summarized in tables and narrative interpretation provided, with particular emphasis on methodological heterogeneity and outcome measures.

### Confidence in cumulative evidence

The revised and validated methodological index for non-randomized studies (MINORS) criteria [63] will be used to assess the strength of non-randomized studies included for the review. The MINORS tool applies a scoring system across 12 items to assess the methodological and scientific value of studies, with the first 8 items relating to non-comparative studies and all 12 items relevant for comparative studies. Each item will be scored from 0 to 2, with 0 indicating a lack of reporting of the item, 1 indicating inadequate reporting and 2 indicating adequate reporting of the item in the evaluated study with maximum scores for non-comparative and comparative studies of 16 and 24, respectively. The MINORS score for non-randomized studies will be categorized as per 0 < MINORS score < 6 to indicate a very low quality evidence, 6 ≤ MINORS score < 10 to indicate low quality of evidence, 10 ≤ MINORS score < 14 to indicate fair quality of evidence and MINORS score > 15 to indicate good quality of evidence. Where randomized controlled trials are included, in the context of a primary comparison between alternative interventions with respect to the review outcomes, the Grading of Recommendations Assessment, Development and Evaluation (GRADE) system will be utilized to assess study quality [58]. For preclinical evidence, the methods proposed by Hooijmans et al. [64] will be used to rate the quality of evidence against the Animal Research: Reporting of In Vivo Experiments (ARRIVE) guidelines for animal research [65].

## Discussion

The results of this review will be published in relevant scientific journals or presented at national or international conferences (‘publications’) by the Investigators.

### Documenting protocol amendments

Protocol amendments and updates will be documented via PROSPERO online register. The nature of the changes made will be recorded, dated and accessible along with the most recent version within the record audit trail under the systematic review protocol registration number CRD42018091763.

## Additional file

Additional file 1: Screening and core dataset (CDS) template for Systematic Review. (XLSX 466 kb)

## Abbreviations

ARRIVE: Animal Research: Reporting of In Vivo Experiments
GRADE: Grading of Recommendations Assessment, Development and Evaluation
HHGS: Histological-Histochemical Grading system
ICRS: International Cartilage Regeneration and Joint Preservation Society
KIMRISS: Knee Inflammation MRI Scoring System
KL: Kellgren-Lawrence
KOOS: Knee injury and Osteoarthritis Outcome Score
KPS: Knee Pain Scale
MeSH: Medical subject headings
MINORS: Methodological index for non-randomized studies
MOAKS: MRI Osteoarthritis Knee Score
MRI: Magnetic resonance imaging
MSC: Mesenchymal stem/stromal cell
OA: Osteoarthritis
OARSI: Osteoarthritis Research Society International
OAS: Oswestry Arthroscopic Score
PICOS: Population, Intervention, Comparator, Outcomes, Study design
PRISMA: Preferred Reporting Items for Systematic Reviews and Meta-Analyses
PROSPERO: International prospective register of systematic reviews
RCT: Randomized clinical trial
ROBINS: Risk Of Bias In Non-randomised Studies - of Interventions
SYRCLE: Systematic Review Centre for Laboratory Animal Experimentation
VAS: Visual analogue scale
WOMAC: Western Ontario and McMasters Universities Osteoarthritis Index
WORMS: Whole-Organ Magnetic Resonance Imaging Score
